# Quantitative ventricular trabeculation assessment in cardiac MRI: optimised blood-pool segmentation, box-counting fractal analysis and non-fractal measurements

**DOI:** 10.1007/s10554-026-03687-9

**Published:** 2026-05-15

**Authors:** Jan Sedlacik, Kathryn A. McGurk, Paweł F. Tokarczuk, Ben Statton, Alaine Berry, Massimo Marenzana, Declan P. O’Regan

**Affiliations:** 1 Medical Research Council, Laboratory of Medical Sciences, London, UK; 2 Robert Steiner MR Unit, Mansfield Centre for Innovation, Institute of Clinical Sciences, Imperial College London, Hammersmith Hospital Campus, Du Cane Road, London, W12 0HS UK; 3 National Heart and Lung Institute, Imperial College London, London, UK

**Keywords:** Level-set, Box-counting, Fractal dimension, Convexity, Boundary length ratio, Trabeculated ventricular mass

## Abstract

Quantitative assessment of the ventricular trabeculation by fractal dimension (FD) involves complex processing steps which may impact the results. We optimised the automated processing workflow for a reliable assessment of the left and right ventricles at end-diastole and end-systole which is suitable for the automated analysis of large-scale cohorts. Ventricular trabeculae and blood were segmented using a level-set method optimised to exclude pixels outside the heart on short-axis cardiac MRI. FD was derived by box-counting the trabeculae/blood boundary while investigating the impact of box size, sampling and rotation. Alternative non-fractal measures – the convexity related boundary length ratio (BLR) and the trabeculated mass ratio (TMR) – were also investigated.FD values with and without optimisation showed a strong linear correlation (R^2^ = 0.81) and narrow agreement limit (1.96·SD = 0.063) only for the end-diastolic left ventricle. Linear correlation and agreement was good between the optimised FD and BLR values for both ventricles and cardiac phases (R^2^ = 0.70–0.92, 1.96·SD = 0.037–0.064) but not for TMR (R^2^ = 0–0.37, 1.96·SD = 0.16–1.4). FD, BLR and TMR differed significantly (p < 0.001) between end-diastole and end-systole with lower FD (-0.07 ± 0.06) but higher BLR (0.31 ± 0.25) and TMR (0.26 ± 0.13) values at end-systole.The previously used fractal analysis is suboptimal except for assessing the end-diastole left ventricle. The optimised fractal analysis is suitable for the left and right ventricle at end-diastole and end-systole. The easy to compute non-fractal BLR gives equivalent information like FD. The volume-based TMR, on the other hand, captures different features of the trabeculation.

**Supplementary Information:**

The online version contains supplementary material available at 10.1007/s10554-026-03687-9.

## Introduction

The inner surface of the ventricles of the human heart is not smooth but consists of a complex network of muscular strands, which may play a role in achieving efficient cardiac performance through force transmission and modifying flow dynamics [1]. In recent studies, trabeculation has been associated with genes regulating myocardial contractility, ventricular development and haemodynamic phenotypes [2, 3].

Quantitative phenotyping of trabeculae is challenging due to their complex structure and inter-individual variation. One approach is to assess their irregular structure using fractal dimension (FD). Please also see Supplementary Table S1 for a more detailed definition of the used technical terms. FD can be useful for describing anatomical structures with complex irregular shapes that may not be fully characterised by traditional geometric or morphometric measures [4]. As these structures are not truly fractal, their measurement requires an assessment of the most appropriate implementation of the FD calculation [5]. Nevertheless, recent studies already using FD to assess the left ventricular trabeculation found higher FDs for dilated cardiomyopathy as compared to healthy subjects [2] and a strong association between FD and the end-diastole left ventricular volume in dilated cardiomyopathy [3]. Therefore, FD may be a useful parameter with clinical relevance for assessing the ventricular trabeculation.

Current software tools for assessing trabeculation rely on cardiac magnetic resonance (CMR) imaging. These tools use a series of automated processing steps for detecting the trabecular boundary within the ventricular blood pool and apply a box-counting method to calculate FD. These approaches scale well to large datasets [6–8] but the most appropriate implementation of these processing steps has not yet been assessed. Optimising these processing steps could reduce the variability of the analysis or improve the assessment of more challenging geometries like the right ventricle and left ventricle at end-systole.

Here, we investigate the most appropriate steps in the FD analysis to standardise its calculation and to implement a robust assessment of trabecular complexity in large biobank cohorts. We have also determined how FD analysis could be generalised to biventricular analysis, including both end-diastolic and end-systolic phases. Finally, we have compared FD analysis with alternative parameters assessing the trabeculation that include the convexity-related boundary length ratio (BLR) [9] and the volume-based trabeculated mass ratio (TMR) [10].

## Materials and methods

### Data set

**Fig. 1:**
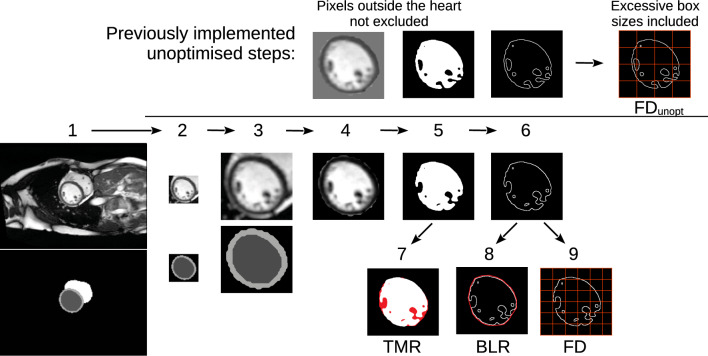
Image processing overview. The upper part denotes the previously implemented unoptimised image processing steps. **1**: Load image slice and corresponding segmentation labels. **2**: Crop image and labels to selected ventricle + 10 mm on each side. **3**: Interpolate cropped image and segmentation labels to 0.25 × 0.25 mm pixel size. **4**: Adjust image pixel intensity excluding pixels outside segmentation labels. **5**: Level-set segmentation of blood pool ignoring outside pixels. **6**: Contour of blood pool segment using Sobel edge detection. **7**: Calculation of Trabeculation Mass Ratio (TMR) using blood pool segment and ventricle label area. **8**: Calculation of Boundary Length Ratio (BLR) using length of blood pool segment and ventricle label contours. **9**: Calculation of Fractal Dimension (FD) of the blood pool contour using the optimised box-counting method

Ninety participants were randomly selected for analysis from the UK Biobank (National Research Ethics Service, 11/NW/0382; application 40616) [11]. The study was not designed to investigate any association of the trabeculation with respect to disease or risk factors. Such studies will be conducted in the future using the optimised trabeculation assessment presented in the paper. The small randomly selected data set was used to investigate the technical aspects of the optimisation. All participants provided written informed consent as part of the UK Biobank recruitment process. End-diastolic and end-systolic CMR images of the short axis of the heart were labelled using the open-source UKBB Cardiac Toolbox (https://github.com/baiwenjia/ukbb_cardiac) [12] employing convolutional neural networks for automated segmentation. The resulting left ventricle (LV), right ventricle (RV), and LV myocardium labels were used for subsequent fractal analysis. Analysis was implemented in MATLAB (R2020b, MathWorks Inc., Natick, Massachusetts, US). Please see Supplementary Table S2 for participant demographics and Supplementary Table S3 for CMR details [13] and Fig. 1 for a depiction of the image processing pipeline. The UK Biobank CMR imaging protocol is reported in detail by Petersen et al. 2016 [13].

### Optimisation of FD processing

Each image slice was cropped to the maximum extent of the analysed LV/RV label plus 10 mm in all directions to include the adjacent myocardium. It is not necessary to include the complete myocardium for the analysis, since the adjacent myocardium is mainly responsible for the contrast of the trabeculae. We do not expect this rough cropping step to cause additional variability of the FD analysis because the original ventricle and myocardium labels are preserved. The cropped images were interpolated to 0.25 × 0.25 mm pixels using the MATLAB *imresize* function with bicubic interpolation. This particular interpolated image resolution was chosen to be consistent with previous studies [2, 3]. Then, the pixel intensities within the heart were normalised and windowed to the range 0–255, with the lowest 1% of the darkest pixels clipped to 0 and the upper 1% of the brightest pixels to 255. This optimisation excludes pixels in the lungs and fat surrounding the heart which are much darker or brighter than the myocardium or blood pool, respectively. Then, the level-set method [14] was used to segment the blood pool and trabeculae within the ventricle label.

To improve the bias-field estimation of the level-set method, we used the *nanconv* function [15] and assigned NaN (Not-a-Number) values to all pixels outside of the investigated ventricle and myocardium labels. This approach ensures that the bias-field estimation is only sensitive to the pixels inside the labels, whereas the result of the previously used standard MATLAB *conv* function is determined by the whole image including all pixels outside of the ventricle and myocardium labels. The outline of the resulting blood pool segment was then obtained by the MATLAB *edge* function using the Sobel edge-detection. These particular level-set segmentation and edge-detection methods were chosen to be consistent with previous studies [2, 3].

The Hausdorff distance (HdD) [16] was calculated between the contours of the segmented blood pool before and after optimisation. The lower the HdD, the better the contours coincide, with the aim being to minimise the variability in these contours due to suboptimal intensity adjustment and bias-field estimation. Furthermore, a visual inspection of the image output before and after optimisation was conducted by the first author (JS) to find obvious failures of the trabeculation assessment. JS has 20 years of experience with MR image analysis in general and 2 years with analysing the cardiac trabeculation in particular. Failures were marked for obvious errors in the segmentation or the contouring of the ventricle or trabeculae. Please see Supplementary Figures S7 and S8 for image examples.

Constructed circular and elliptical contours were used to test the implementation of the box-counting method. Since these are simple closed curves, the mathematically exact FD is 1. Foroutan-pour et al. [17] recommend that the largest box-counting size to not exceed 25% of the shorter side of the image: “Larger box sizes will lead to very poor information and increase the risk of error in many cases.” The selection of the minimum box-counting size is also crucial, since below a certain threshold, the method will simply measure an object’s individual linear elements rather than the global fractal characteristics. Foroutan-pour et al. also suggest that the box-counting sizes must be carefully adjusted for each image individually to obtain the true FD [17]. This is, however, impractical for an automated script and since the ventricular trabeculation is not truly fractal, we used some discretion in calculating FD by the box-counting method. We chose the smallest box-counting size of 2 pixels and the largest size of 25% of the shorter image side. The MATLAB function *polyfit* was used to assess the negative slope of the double logarithmic curve of the box size vs. the box count which corresponds to FD. Although no true FD measurement can be achieved with this box-counting implementation, it is sensitive to differences in the complexity of the trabeculation. We further investigated the effects of different box sampling offsets [18] and object rotations [19] on the FD calculation. Previous works [6, 7] only computed a small subset of possible object samplings, i.e., flipping and rotation by 180° for centred objects (Suppl.Fig.S5, image samplings 9–12). We expanded this to include objects placed on the image borders (Suppl.Fig.S5, image samplings 1–8). We also averaged the estimated FDs for rotations between 0–90° in 5° steps to reduce the variability of the box-counting FD measurement still further.

### Alternative assessment parameters

Due to the complexity of the box-counting method, we also implemented simpler measurements for assessing the ventricular trabeculation. One common parameter in shape analysis is *convexity –* the ratio of the area or contour length of the convex hull and of the actual shape [9]. We modified Definition 3 in [9] to use the ventricle label instead of the convex hull. The boundary length ratio (BLR) was calculated by:

1 $$\mathrm{BLR}=\frac{\mathrm{length}\left(\text{ventricle labe}\text{l contour}\right)}{\mathrm{length}\left(\text{blood pool contour}\right)}$$

This convexity-based ratio is higher for smooth convex shapes and lower for more complex blood pool contours. The length of more complex contours is longer and, thus, reducing the ratio.

Another parameter assessed was the trabeculated mass ratio (TMR), which is used regularly to assess ventricular trabeculation [10, 20]. We calculated the TMR by:

2 $$\mathrm{TMR}=1-\frac{\mathrm{area}\left(\text{blood pool segment}\right)}{\mathrm{area}\left(\text{ventricle label}\right)}$$

which treats all non-blood areas within the ventricle label as trabeculated mass regardless of the complexity of the blood pool boundary.

### Statistical analysis

Linear regression models were fitted between the different trabeculation parameters using the *lmfit* function of MATLAB. Bland–Altman plots and limits of agreement (1.96·SD) were calculated between the differences of the estimates of the linear model and the optimised FD values. We also compared the optimised quantitative trabeculation parameters obtained at end-diastole and end-systole using a paired t-test.

## Results

### Optimisation of FD processing

**Fig. 2:**
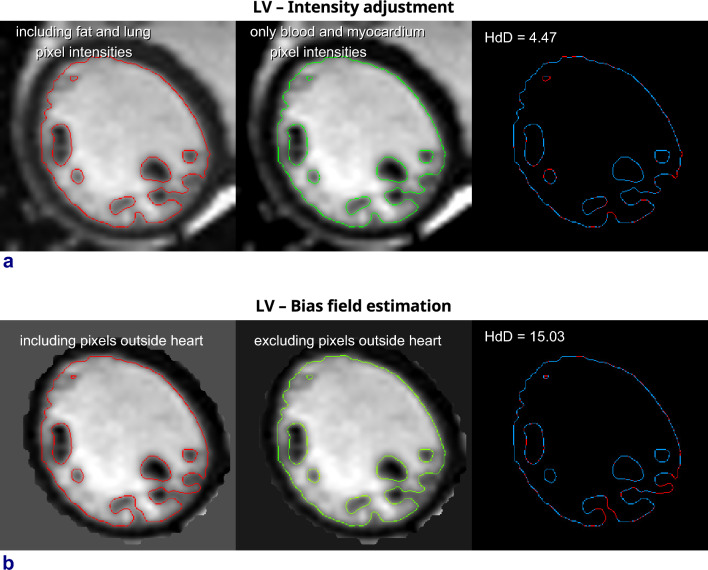
Impact of intensity adjustment (**a**) and bias-field estimation (**b**) including or excluding outside pixels on the blood pool segmentation of the level-set method. Red: Segment outlines of unoptimised code. Green: Segment outlines of optimised code. Blue: Overlapping segment outlines. HdD: Hausdorff distance. The differing contours demonstrate that pixels outside the heart affect the blood pool segmentation which impacts the subsequent trabeculation assessment and may increase its variability which we aim to minimise. Impact on right ventricle see Supplementary Figure S1

**Fig. 3:**
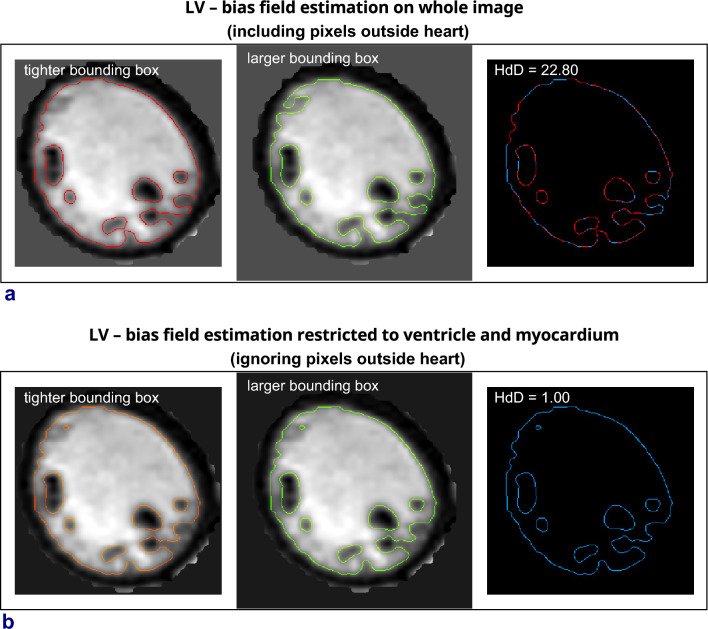
Impact of different image bounding box sizes on the blood pool segmentation of the unoptimised (**a**) and optimised (**b**) level-set methods. Red: Segment outlines resulted from tighter bounding box. Green: Segment outlines resulted from larger bounding box. Blue: Overlapping segment outlines. HdD: Hausdorff distance. The optimised code results in nearly identical contours regardless of the number or intensities of pixels outside the heart. Impact on right ventricle see Supplementary Figure S2

The contours of the end-diastole LV trabeculae (Fig. 2) obtained with the optimised pixel intensity scaling or bias-field estimation differed from the previously unoptimised implementation with HdD = 4.47 and HdD = 15.03, respectively. Furthermore, the optimised bias-field estimation resulted in nearly identical contours (HdD = 1.0) regardless of the image bounding box size (Fig. 3). The RV segmentation shows similar effects regarding the optimised pixel intensity scaling and bias-field estimation (Suppl.Figs.S1-S2).

**Fig. 4:**
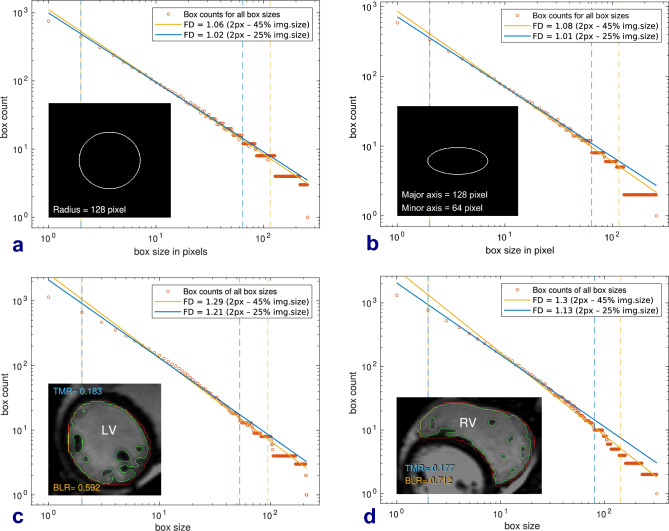
Box counts and fractal dimensions (FD) derived from the slope of the linear fits between the box sizes of 2 pixels and 25% (blue lines) or 45% (yellow lines) of the image size. The vertical blue and yellow dashed lines denote the corresponding box size limits. The inset images show the analysed contours (**a**: circle, **b**: ellipse, **c**: left ventricle (LV) green contour and d: right ventricle (RV) green contour). Reducing the largest box size to 25% of the image size gives FD values closer to the expected value of 1.0 for the analysed test contours (**a, b**) and lower FD values for the LV and RV example contours (**c, d**) as compared to the previously used 45% box size. The contours of the ventricle label (red) and the boundary length ratio (BLR) and trabeculated mass ratio (TMR) are also shown for the LV and RV (**c, d**)

The box-counting FDs of the circular/ellipsoidal test contours are close to 1 when setting the largest box-counting size to 25% of the smallest image side. The previously implemented larger box-counting size of 45% [7] gives higher FD values (Fig. 4, upper row). Furthermore, the FD values for the example ventricles are also lower with the optimised largest box-counting size of 25% as compared to 45% (Fig. 4, lower row). Additional optimisation results regarding object size, sampling and rotation are given in the supplementary materials (Suppl.Figs.S3-S6).

### Regression and agreement analysis

**Fig. 5:**
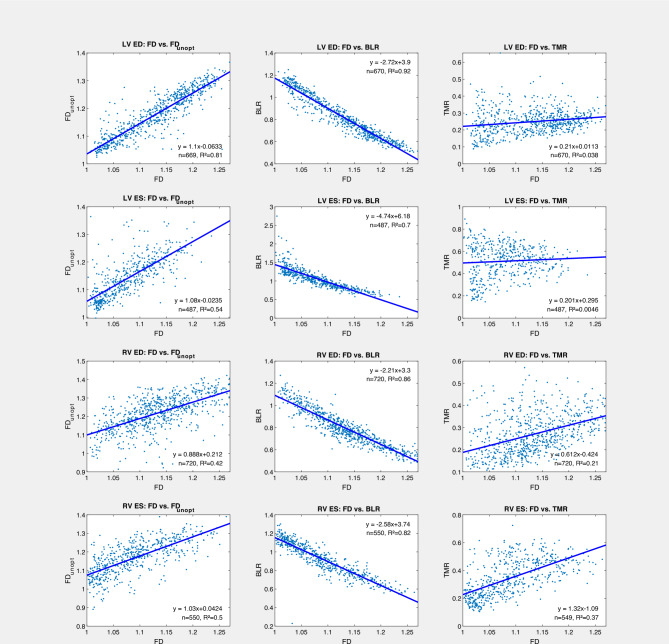
Scatter plots and linear regression of fractal dimension (FD) values of the optimised code vs. the unoptimised code (FD_unopt_) as well as the alternative trabeculation estimates BLR and TMR of 90 randomly selected UK Biobank participants. Top row: end-diastolic left ventricle (LV); Second row: end-systolic LV; Third row: end-diastolic right ventricle (RV) and Bottom row: end-systolic RV. The corresponding Bland–Altman plots are shown in Fig. 6. The analysed image data of the example outlier points marked by a green, magenta and orange circle is shown in Supplementary Figure S7

**Fig. 6:**
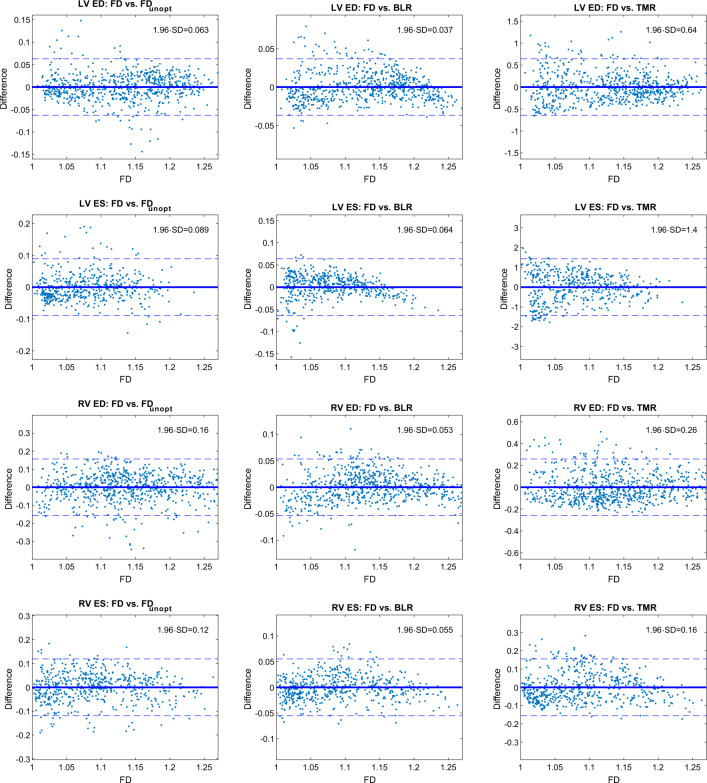
Bland–Altman plots and limits of agreement (1.96·SD) of the linear regressions of fractal dimension (FD) values of the optimised code vs. the unoptimised code (FD_unopt_) as well as the alternative trabeculation estimates BLR and TMR shown in Fig. 5. Top row: end-diastolic left ventricle (LV); Second row: end-systolic LV; Third row: end-diastolic right ventricle (RV) and Bottom row: end-systolic RV

The regression and agreement analysis between the FD values of the unoptimised code (FD_unopt_) and the optimised code (FD) show a good linear correlation (R^2^ = 0.81) and narrow limits of agreement (1.96·SD = 0.063) for the end-diastolic LV. However, the more challenging shapes of the end-systolic LV and the RV show inferior fits and larger agreement limits between the previous and optimised FDs (R^2^ = 0.42–0.54, 1.96·SD = 0.089–0.16) (Figs. 5 and 6, left column). BLR fits well with the optimised FD (R^2^ = 0.70–0.92), showing narrow limits of agreement (1.96·SD = 0.037–0.064) for all data sets (Figs. 5 and 6, middle column), suggesting an additional validation of the optimised FD. The fits between TMR and FD are poor (R^2^ = 0.00–0.37), with large limits of agreement (1.96·SD = 0.16–1.4) (Figs. 5 and 6, right column), suggesting that TMR captures a different feature of the trabeculae than its complexity.

### End-diastole/systole comparison

**Fig. 7:**
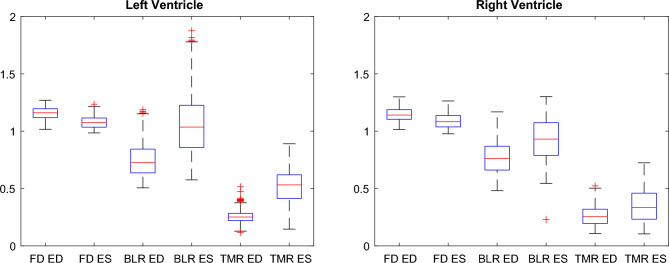
Box plots of all left- and right-ventricular (LV/RV) quantitative trabeculation parameters for end-diastole/end-systole (ED/ES) comparison. Please see corresponding numerical results in Table.1

All three parameters FD, BLR and TMR showed statistically highly significant (p < 0.001) differences between the end-diastole and end-systole phase for both ventricles. FD is lower at end-systole as compared to end-diastole, whereas BLR and TMR are higher at end-systole (Table 1 and Fig. 7).

**Table 1:** Comparison of left- and right-ventricular (LV/RV) quantitative trabeculation parameters between end-diastole and end-systole (mean values ± standard deviation)

	End-diastole	End-systole	Difference
LV-FD	1.15 ± 0.05	1.08 ± 0.05	−0.07 ± 0.06 (p < 0.001)
LV-BLR	0.76 ± 0.16	1.06 ± 0.28	0.31 ± 0.25 (p < 0.001)
LV-TMR	0.26 ± 0.05	0.51 ± 0.15	0.26 ± 0.13 (p < 0.001)
RV-FD	1.15 ± 0.06	1.09 ± 0.06	−0.06 ± 0.05 (p < 0.001)
RV-BLR	0.77 ± 0.15	0.92 ± 0.17	0.15 ± 0.14 (p < 0.001)
RV-TMR	0.26 ± 0.08	0.35 ± 0.13	0.08 ± 0.09 (p < 0.001)

Please see corresponding box-plots in Fig. 7

### Segmentation failures

Visual inspection showed low failure rates of 0.7% and 0.1% in all end-diastolic LV images for the previous and the optimised code, respectively. The failure rate of the previous code was 8.8%, 12.5% and 16.9% for the end-systolic LV, end-diastolic RV and end-systolic RV, respectively. The optimised code showed much lower failure rates below 1% for all of these (Suppl.Tab.S4). Image examples of the segmentation failures are shown in Suppl.Fig.S7 and S8.

## Discussion

We demonstrated that improvements to the level-set method by excluding pixels outside the ventricle and myocardial labels reduce the variability of the blood pool segmentation. Optimising the box-counting method by reducing the largest box-counting size, adding more sampling options and averaging the FD over different object rotations, further reduces the variability of the FD calculation. Alternative measures for assessing ventricular trabeculation seem to give equivalent (BLR) or different (TMR) information with respect to FD.

The current approach for assessing ventricular trabeculation is limited by the quality of the ventricular and myocardial labels [12]. Furthermore, the implemented level-set method [14] does not discriminate between trabeculae and papillary muscles, which are subsequently included in the assessment and could increase variability. We chose the particular labelling and level-set methods to be consistent with previous FD studies [2, 3]. The crucial optimisation step was to make the segmentation insensitive to pixels outside of the ventricle and myocardium labels which would also apply to any other labelling or segmentation method. An alternative labelling method which would also detect and label the papillary muscles [20] could be used to exclude the papillary muscles from the trabeculation assessment which will affect the derived quantitative parameters. Therefore, trabeculation parameters can only be compared within consistently labelled and segmented data sets. Excluding the papillary muscles might reduce the variability of the trabeculation assessment. However, including the papillary muscles might increase its sensitivity, since the morphology of the papillary muscles is a clinical feature that is observed alongside increased trabecular complexity.

The performance of the optimised fractal analysis has been carried out using images of healthy UK Biobank participants scanned with a highly standardised CMR imaging protocol. Strongly abnormal trabeculation or variations of the CMR imaging protocol were not tested in the presented work. However, the FD parameter derived by the previously implemented unoptimised code found higher FDs for dilated cardiomyopathy as compared to healthy subjects [2] and a strong association between FD and the end-diastole left ventricular volume in dilated cardiomyopathy [3]. Therefore, we are confident that the optimised FD calculation will also be sensitive to abnormal trabeculation in cardiomyopathy. To account for differences in the CMR imaging protocol which might affect the depiction of anatomical details and, therefore, the measured FD, we recommend to control for such effects by taking the different imaging parameters, like different acquisition pixel sizes, into account as co-variates in the subsequent statistical analysis.

The CMR data available to us had no repeated same scan frames or repeated examinations for analysing the test–retest reliability of the derived trabeculation parameters. However, if the depiction of the trabeculae is similar between repeated frames or examinations, we expect the fractal analysis, also, to give similar results. Highly concordant results were found for replication studies conducted by Meyer et al. 2020 [2] using the originally implemented unoptimised fractal analysis tool and genetic data of replication cohorts. This suggests that the fractal analysis part of these replication studies must have had a reasonably good test–retest reliability, which would also apply to the presented optimised fractal analysis.

Reducing the largest box size of the box-counting method excludes the error-prone counts at larger box sizes. This results in lower FD values but also reduces the variability for different sampling options and object rotations. This reduction of the FD variability makes the ventricular trabeculation assessment a more robust trait for future genome-wide association studies. The similar average FDs obtained for object rotations between 0–90° using 1° or 5° steps allow us to reduce the computational costs by implementing rotation steps of only 5° in the optimised code without sacrificing accuracy. The detected increase in the variability of the FD estimates for smaller object sizes, e.g., the circular and elliptical test contours, suggests that the FD values for very small ventricle sizes should be ignored.

The previous, unoptimised code exhibited a low failure rate (as determined by visual inspection of the image output) for the end-diastole LV, but a higher rate of failure in all other cases (i.e., the end-systole LV, or the RV at both phases). The overall low failure rate of the optimised code, for all data sets, suggests that the trabeculation can now adequately be assessed for all cases.

The observed higher FD values of the unoptimised code [6, 7] do not allow for direct comparison with the optimised code FD values. However, the good linear fit and narrow agreement limits between FD and FD_unopt_ for the end-diastolic LV suggest that the previously noted genome-wide associations for the end-diastolic LV [2, 3, 8] are valid. Furthermore, the good fits and narrow limits of agreement between FD and BLR for both ventricles and cardiac phases suggest that BLR is a valid alternative measurement for assessing ventricular trabeculation without the need for the complex box-counting method. Also, the poor fits and large agreement limits between FD and TMR suggest that the volume-based TMR captures a different feature of the ventricular trabeculation which may complement the boundary-based FD or BLR measurements. We also expect that other volume derived cardiac parameters, like the blood volume and myocardial mass and their ratios (myocardial contraction fraction), will show a similarly low agreement with FD, since FD assesses the complexity of the trabeculated boundary layer and is not a volumetric measurement.

The observed reduction of the FD at end-systole is in agreement with previous work investigating the change of the FD over the cardiac cycle of the left ventricle using CT data [21, 22] or the right ventricle using CMR data [23]. The opposed effect of increased BLR at end-systole is explained by the negative correlation of BLR with FD. Whereas, the increased TMR is caused by the much more decreased blood volume at end-systole but less reduced volume of the trabeculated mass due to the limited compressibility of the myocardium [24]. Furthermore, the observed variability of the FD measurements within the test data set is consistent with Kawakubo et al. 2019 [23] who measured FD on cardiac MR cine images, as in the presented work.

In conclusion, the previously used fractal analysis tool is limited for assessing the trabeculation of the left ventricle at end-diastole. The optimised fractal analysis tool is suitable for assessing the left and right ventricles at end-diastole and end-systole. Furthermore, the easily computed non-fractal BLR parameter gives similar information to the FD measurements, whereas the volume-based TMR measure captures different features of the trabeculation.

## Supplementary Information

Below is the link to the electronic supplementary material.

Supplementary file2

## Data Availability

The dataset supporting the conclusions of this article is included within the article and its supplemental files. CMR data for running the optimised code is provided via GitHub: Project name: AutoFD; Project home page: https://github.com/ImperialCollegeLondon/AutoFD; Operating system: Platform independent; Programming language: Matlab; Other requirements: Matlab version R2020b or higher; License: MIT License; No restrictions to use by non-academics.

## Abbreviations

BLR: Boundary length ratio
CMR: Cardiac magnetic resonance
FD: Fractal dimension
HdD: Hausdorff distance
LV: Left ventricle
NaN: Not-a-number
RV: Right ventricle
SD: Standard deviation
TMR: Trabeculated mass ratio
