# Supplementary material for: Quantitative ventricular trabeculation assessment in cardiac MRI: optimised blood-pool segmentation, box-counting fractal analysis and non-fractal measurements
Source: Int J Cardiovasc Imaging. 2026 May 15;42(7):1369–79. doi: 10.1007/s10554-026-03687-9 (PMC13375774; doi:10.1007/s10554-026-03687-9)
Supplement: Supplementary file 1 — Supplementary file2 [file 10554_2026_3687_MOESM1_ESM.docx]

Quantitative ventricular trabeculation assessment in cardiac MRI: Optimised blood-pool segmentation, box-counting fractal analysis and non-fractal measurements

**Supplementary Materials**

**Supplementary Table S1:** Table of definition of used the technical terms.

| Box-counting method | This method is used to in the fractal analysis of complex shapes or patterns by dividing the image into smaller squared sections of different sizes and counting how many boxes contain part of the shape or pattern. |
| --- | --- |
| Bias-field estimation | This method corrects for intensity inhomogeneities caused by imperfections in imaging devices or objects. This involves estimating and correcting the bias field to improve the accuracy of image analysis and segmentation. |
| Boundary Length Ratio (BLR) | This parameter is a simple way to quantify the complexity of the trabeculation boundary as compared to the fractal dimension. It is calculated by the ratio of the length of the boundary of the ventricle label and the contour of the trabeculae within the ventricle. |
| Fractal analysis and Fractal Dimension | The fractal dimension (FD) is used in fractal analysis to quantify the complexity of a shape or pattern. FD can be determined by the box-counting method by the linear slope of the double-logarithmic plot of the different box sizes vs. the number of boxes containing part of the analysed pattern. |
| Hausdorff distance (HdD) | This parameter can be used to quantify how far two contours are from each other. It is defined as the greatest distance from a point in one contour to the closest point in the other, indicating how similar or dissimilar the two contours are. |
| Level-set method | This method is good at segmenting complex shapes in images with intensity inhomogeneity by incorporating local and global image features. |
| Sobel edge-detection | This image processing technique identifies edges in images by calculating the gradient of the image intensity. |
| Trabeculated Mass Ratio (TMR) | This parameter assesses the extent of the trabeculation in the heart. It compares the mass of trabeculated myocardium to the total myocardial mass, which can provide insights into cardiac function and potential health outcomes. |

**Supplementary Table S2:** Participant demographics.

| **Characteristics** | **Range or n in % or mean ± SD** |
| --- | --- |
| Age at CMR | 47-81 years (median=66 years) |
| Sex | 51.3% female; 42% male; 6.7% had no record |
| Ancestry | 77% British; 23% Other |
| Systolic blood pressure (mmHg) | 142.82 ± 24.72 |
| Hypertension | 36% yes; 64% no |
| Hypercholesterolaemia | 22% yes; 78% no |
| Diabetes mellitus | 3% yes; 97% no |
| Body mass index (kg/m^2^) | 27.02 ± 4.56 |
| Alcohol intake (grams per day) | 13.11 ± 14.08 |
| Vigorous physical activity (days per week) | 2.03 ± 2.29 |
| Moderate physical activity (days per week) | 3.80 ± 2.55 |

**Supplementary Table S3:** Cardiac MR imaging details.

| **Characteristics** | **Details** |
| --- | --- |
| ‍Date of CMR | May 2014 – April 2022 |
| Pulse sequence | Balanced steady state free precession (bSSFP) cine |
| Location/Orientation/Coverage | Base to apex in short-axis views (approximately 10 slices) |
| Flip angle | 80° |
| TE/TR | 1.1/2.6 ms |
| Parallel acquisition acceleration factor | 2 |
| Slice thickness/gap between slices | 8/2 mm |
| Typical Field of View | 380 × 252 mm |
| Matrix size | 208 × 187 |
| Phase encoding direction | Anterior-Posterior but can also be Left-Right depending on patient anatomy |
| Temporal resolution | 31.56 ms |
| Calculated cardiac phases | 50 |
| ECG triggering | Retrospective |
| Image/Raw/Elliptical Filters | Off |
| Distortion Correction | 2D |
| Breath-holds | 1 slice per breath-hold (end-expiration) |

**Supplementary Table S4:** Visual inspection of image output for processing failures. See Supplementary Figures S9 and S10 for image examples.

| **Data set** | **Analysed images** | **Failures of previous code (rate)** | **Failures of optimised code (rate)** |
| --- | --- | --- | --- |
| End-diastolic LV | 669 | 5 (0.7%) | 1 (0.1%) |
| End-systole LV | 487 | 43 (8.8%) | 2 (0.4%) |
| End-diastolic RV | 720 | 90 (12.5%) | 6 (0.8%) |
| End-systole RV | 550 | 93 (16.9%) | 3 (0.5%) |


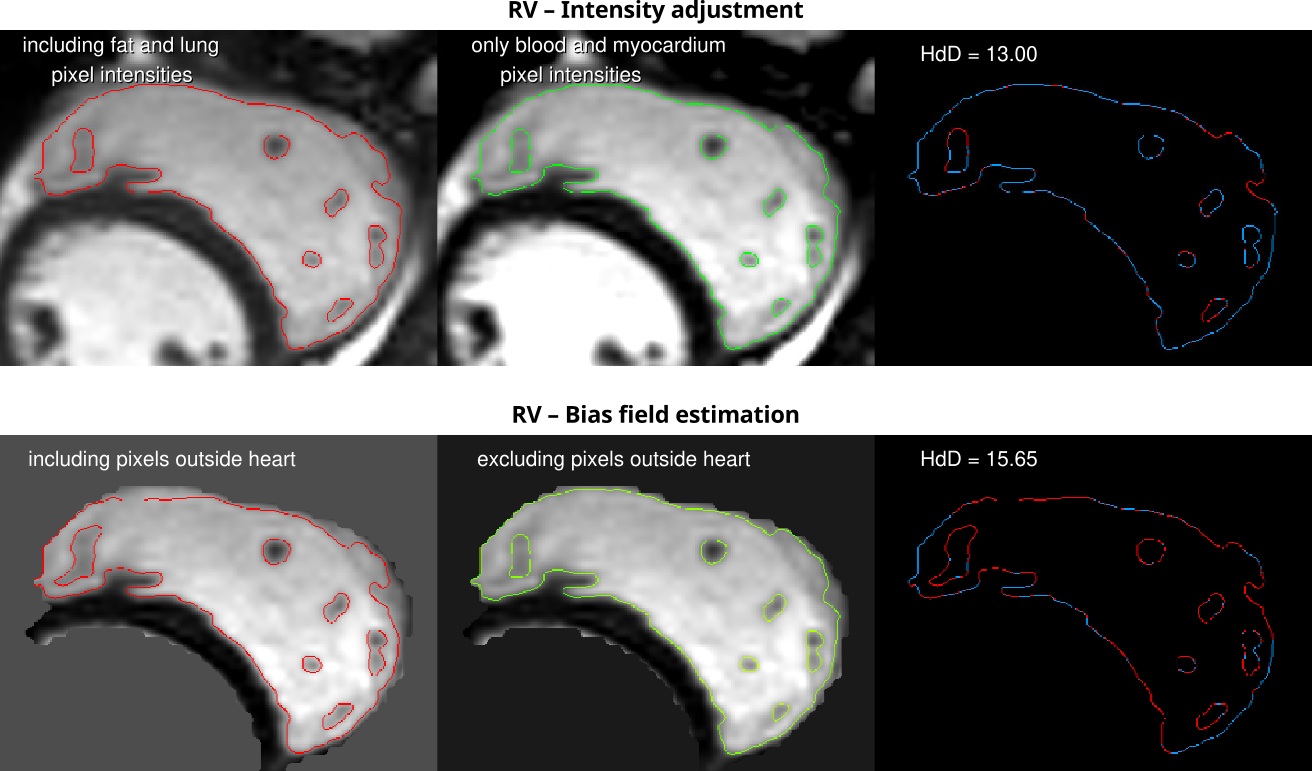
**Supplementary Figure S1:** Impact of intensity adjustment and bias-field estimation including or excluding outside pixels on the blood pool segmentation of the level-set method. Red: Segment outlines of unoptimised code. Green: Segment outlines of optimised code. Blue: Overlapping segment outlines. HdD: Hausdorff distance. The differing contours demonstrate that pixels outside the heart affect the blood pool segmentation which impacts the subsequent trabeculation assessment and may increase its variability which we aim to minimise. Impact on left ventricle see Figure 2.


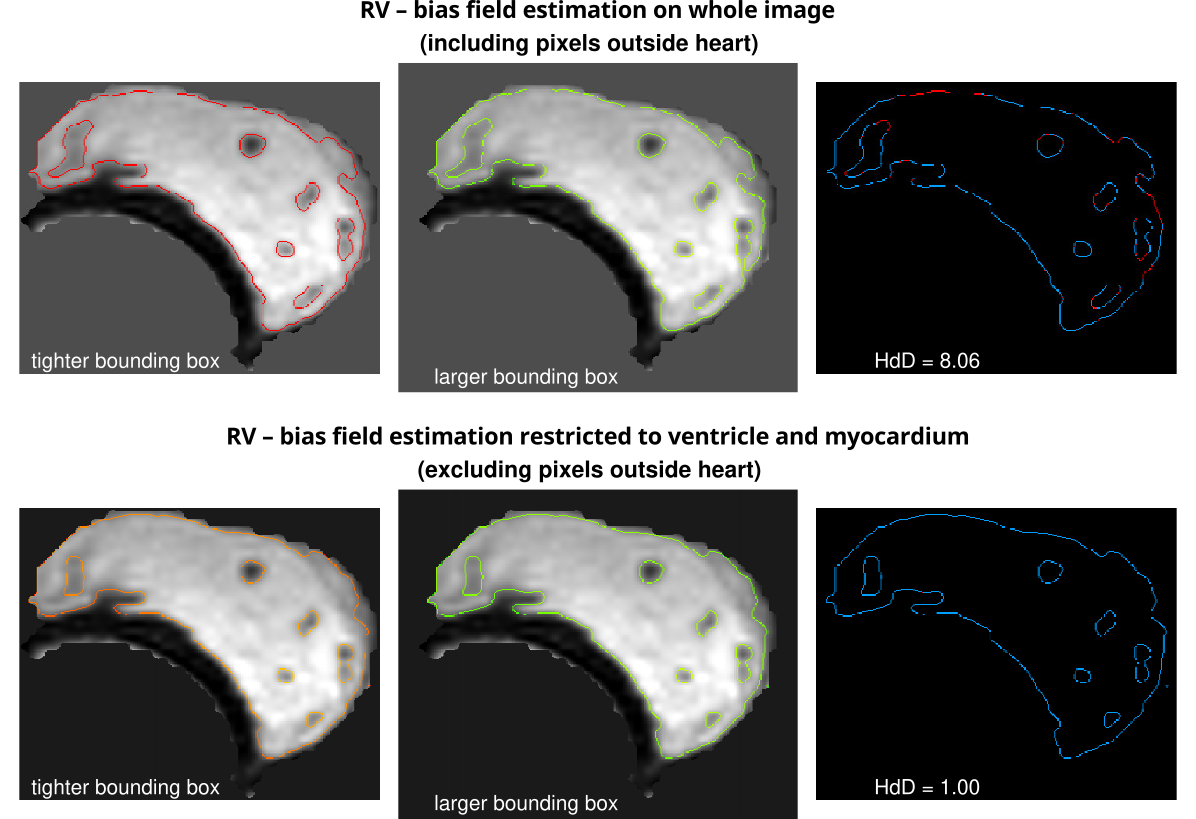
**Supplementary Figure S2:** Impact of different image bounding box sizes on the blood pool segmentation of the unoptimised and optimised level-set methods. Red: Segment outlines resulted from tighter bounding box. Green: Segment outlines resulted from larger bounding box. Blue: Overlapping segment outlines. HdD: Hausdorff distance. The optimised code results in nearly identical contours regardless of the number or intensities of pixels outside the heart. Impact on left ventricle see Figure 3.


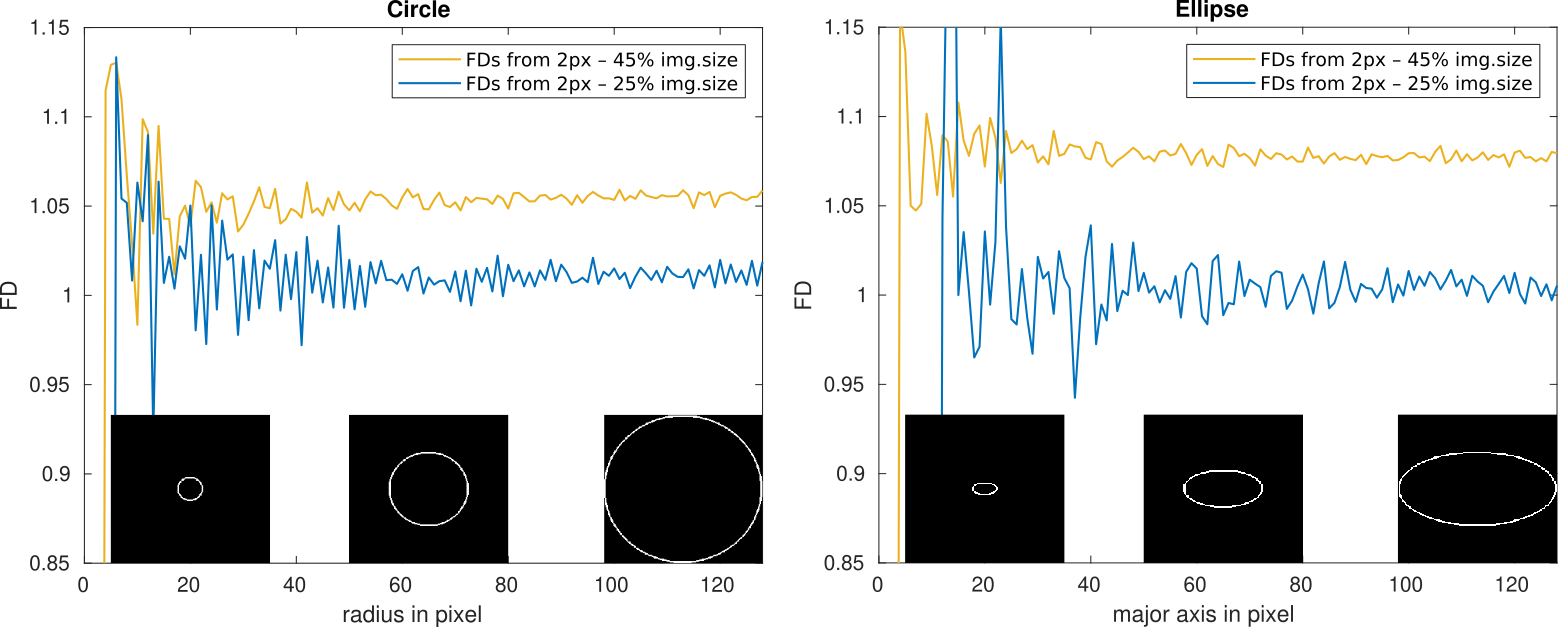
**Supplementary Figure S3:** Impact of circle radius and ellipse size on fractal dimension (FD) calculation of the unoptimised (yellow) and optimised code (blue). The variability of FD increases with smaller radii/sizes and the FDs calculation becomes unreliable below a certain radius/size due to the finite image pixel size.


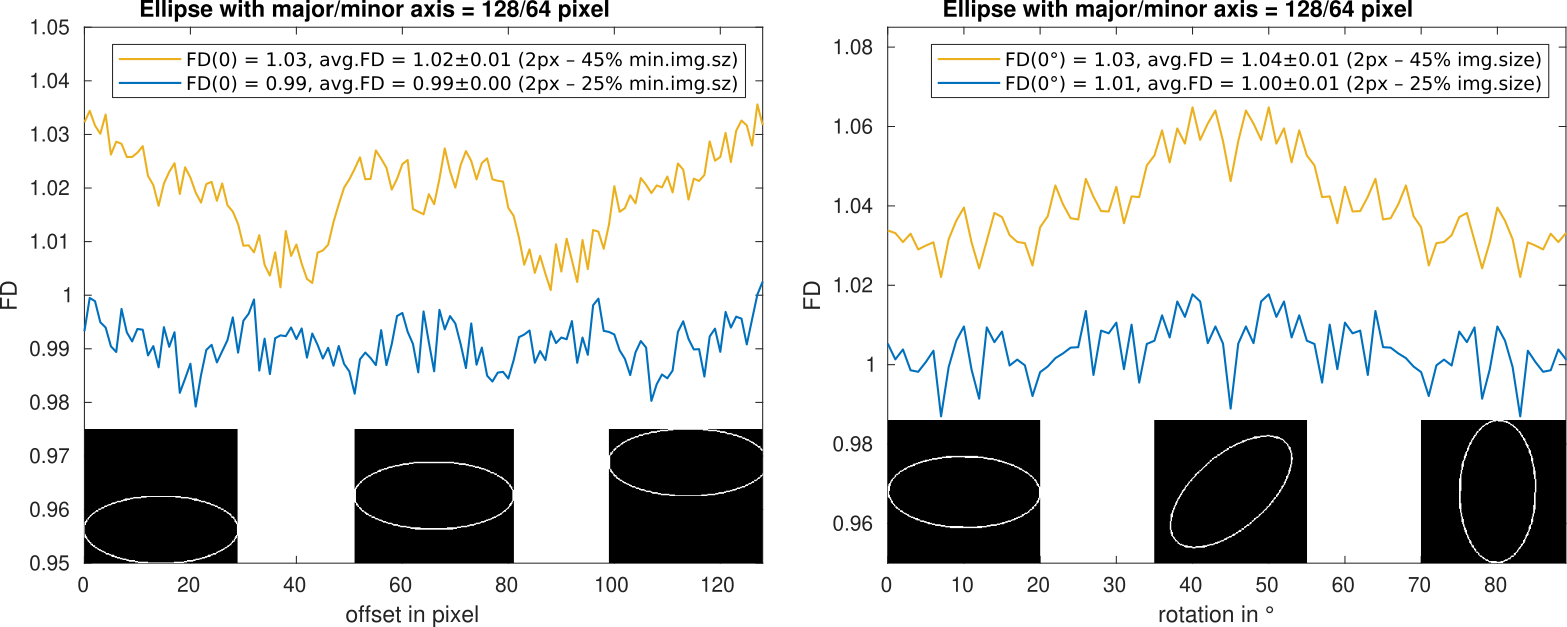
**Supplementary Figure S4:** Impact of ellipse offset (left) and rotation angle (right) on estimated fractal dimension (FD) values of the unoptimised (yellow) and optimised code (blue). The variability of FD is reduced for the largest box size of 25% as compared to the suboptimal 45%.


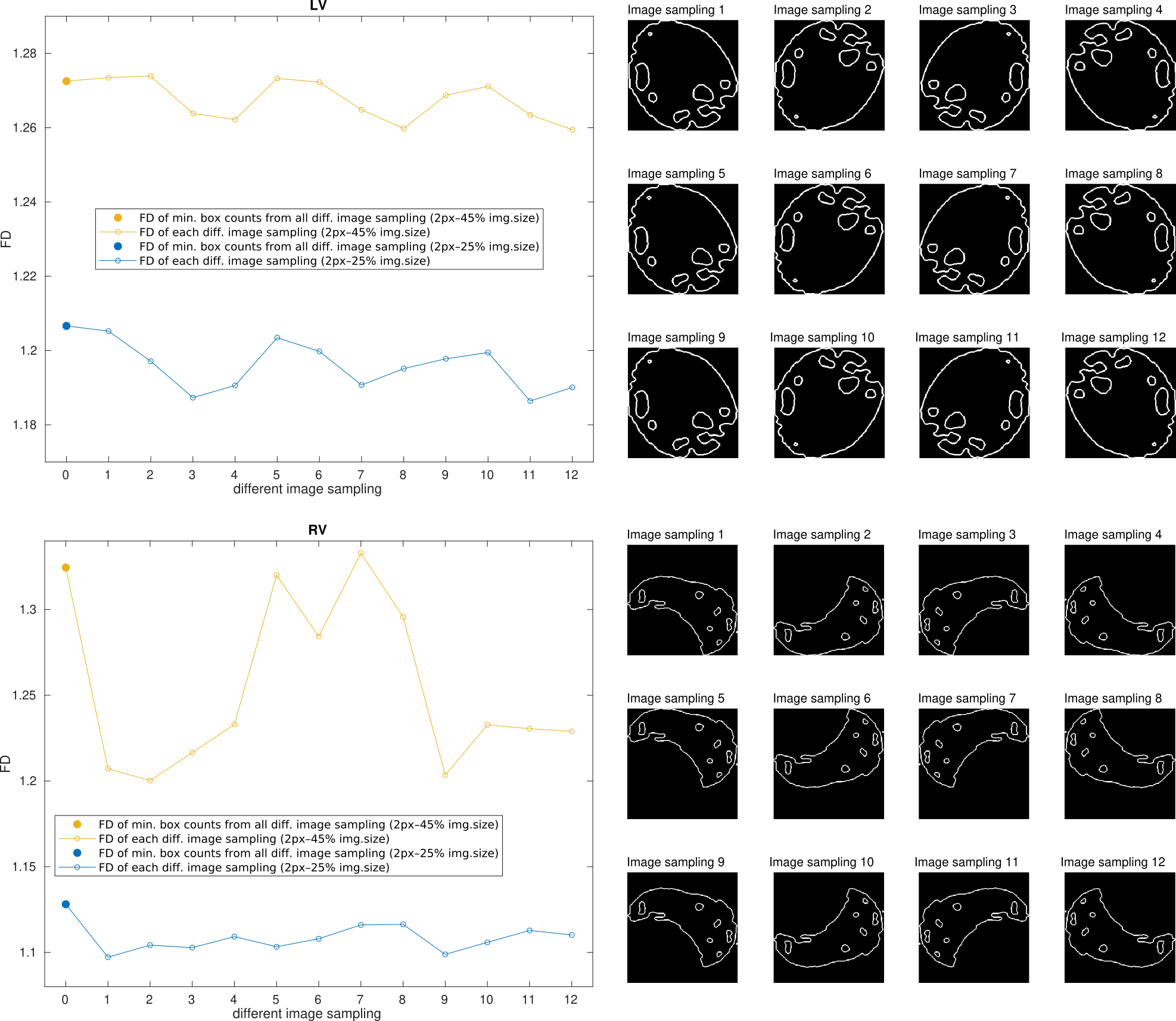
**Supplementary Figure S5:** Impact on estimated fractal dimensions (FD) for different sampling options (padding and flipping) of the left (LV) and right ventricle (RV) example ventricle contours. The variability is lower for the LV and for the optimised code (blue). The estimated FD from the minimum box counts over all different sampling options is highlighted in the plots by a solid circular marker at position 0 and results in a high FD found for only a few sampling options.


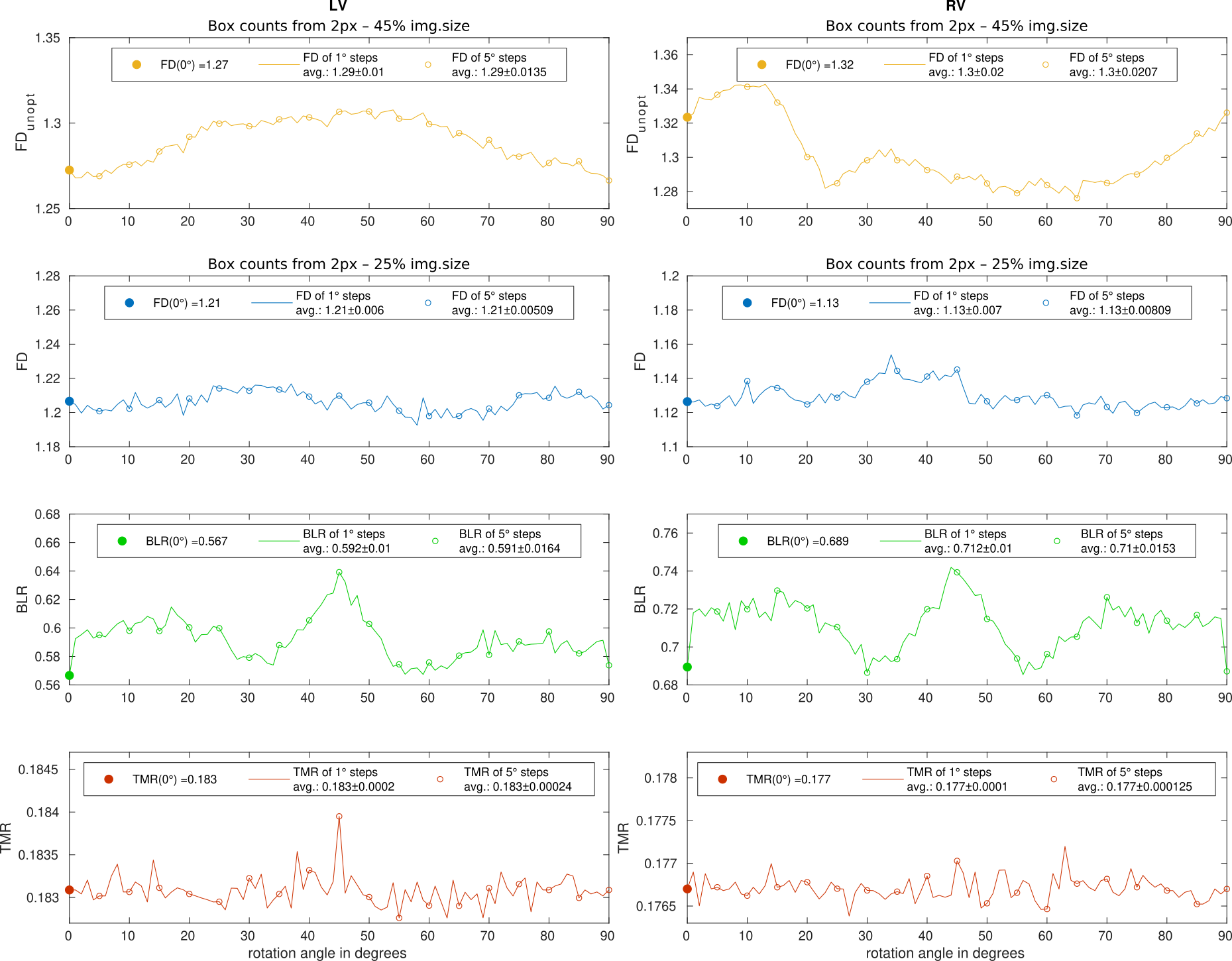
**Supplementary Figure S6:** Dependency of fractal dimension (FD), boundary length ratio (BLR) and trabeculated mass ratio (TMR) on the rotation of the example ventricle segments (Suppl.Fig.S5). The values at 0° are highlighted by a solid circular marker and the numerical values are shown in the legend. The values for each 5° step are marked by a hollow circular marker and the values for 1° steps are represented by the solid lines. The variability of the FD for different object rotations is lower for the optimised code. The BLR shows some variability with respect to object rotation, but the TMR is insensitive to it. Average values and standard deviation over all 1° and 5° steps are shown in the plot legends and are similar.

**Supplementary**
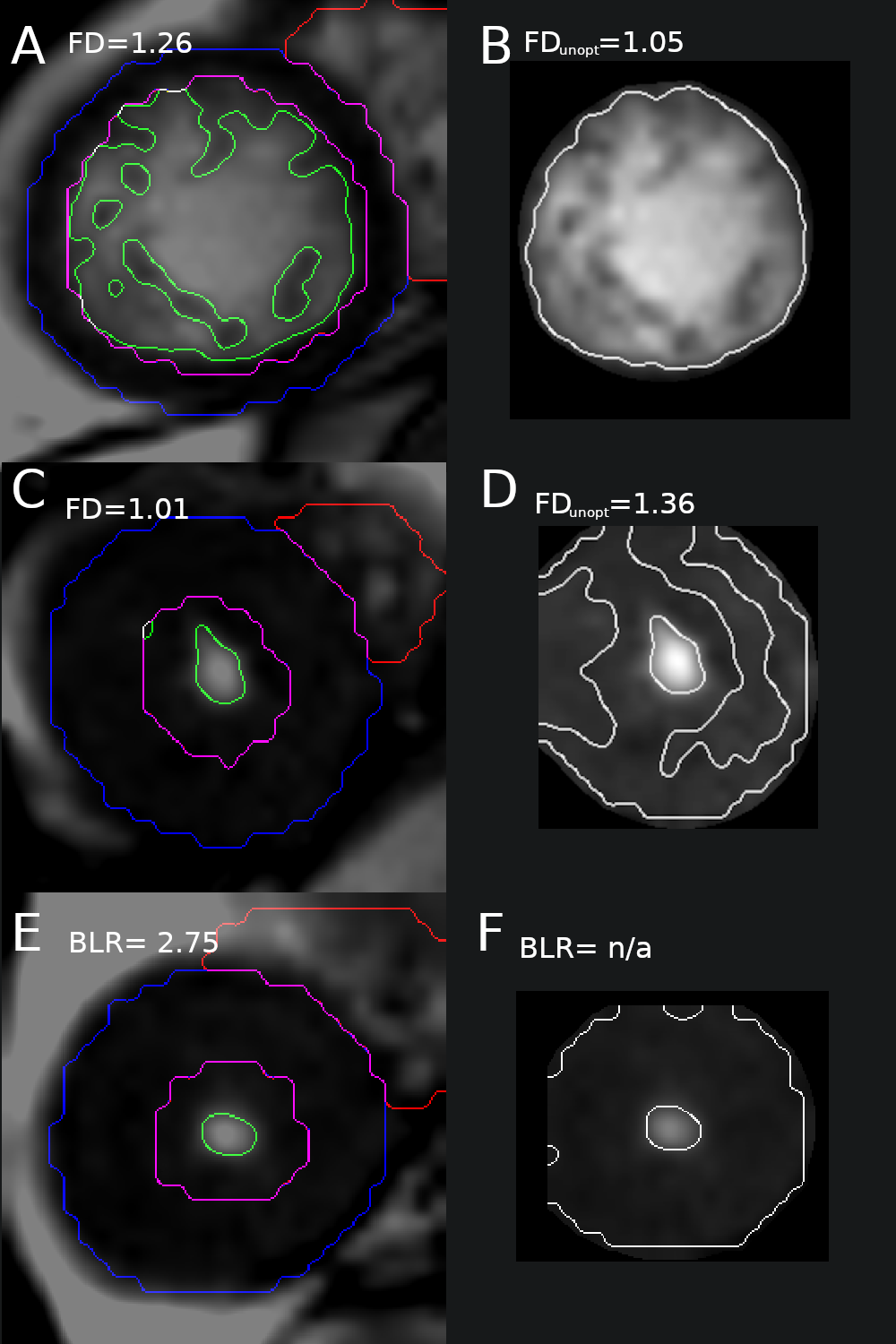
**Figure S7:** Example output image data of the previous and optimised automated FD calculation marked by the green (A and B), magenta (C and D) and orange (E and F) circles in Supplementary Figure S7. **A, C, E:** output of optimised code. Green: trabeculae/blood boundary used for box-counting method; Magenta: left ventricle boundary; Blue: myocardium boundary; Red: right ventricle boundary; Myocardium overlapping with the right ventricle boundary is also magenta. Overestimated ventricle label, e.g., in E causes a high BLR mostly seen in the end systole LV. **B, D, F:** output of previous code. White: trabeculae/blood boundary used for box-counting method. The trabeculae/blood segmentation seems to fail for the previous code which results in too low (B) or too high (D) FD values compared to the optimised code. No Boundary length ratio was calculated by the previous code (F).


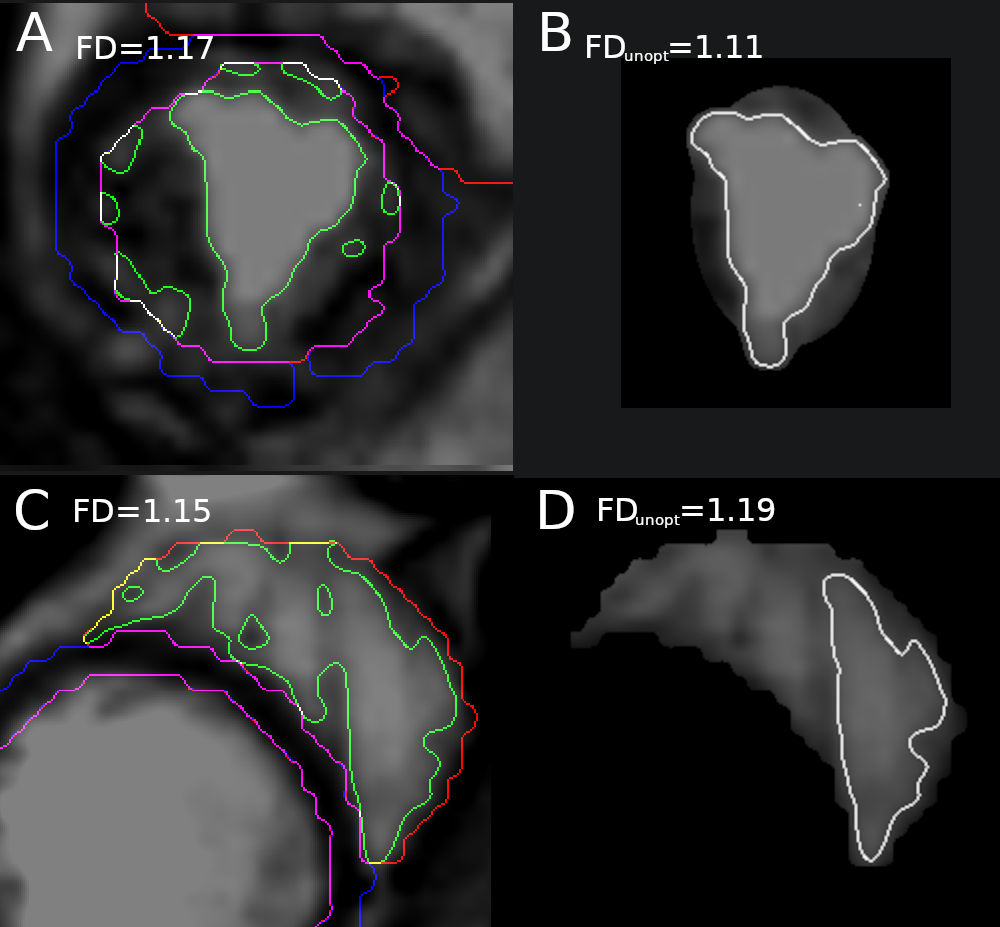
**Supplementary Figure S8:** Example output image data of obviously failed trabeculation assessments. **A and C:** output of optimised code. Green: trabeculae/blood boundary used for box-counting method. Yellow: conforming trabeculae/blood boundary. Magenta: left ventricle boundary. Blue: myocardium boundary. Red: right ventricle boundary. Myocardium overlapping with the right ventricle boundary is highlighted in magenta. **B and D:** output of previous code. White: trabeculae/blood boundary used for box-counting method. The trabeculae/blood segmentation seems to fail for the optimised code in A where the label of the left ventricle includes too much of the myocardium. Furthermore, the segmentation fails for previous code in D where only half of the blood pool is segmented.
